# The case for intraocular delivery of PPAR agonists in the treatment of diabetic retinopathy

**DOI:** 10.1186/1471-2415-12-46

**Published:** 2012-09-02

**Authors:** Maxwell P Treacy, Tara P Hurst

**Affiliations:** 1 Royal Victoria Eye and Ear Hospital, Adelaide Road, Dublin 2, Dublin, Ireland; 2 School of Science and Technology, Nottingham Trent University, Clifton Lane, Nottingham, NG11 8NS, United Kingdom

**Keywords:** Diabetes, Diabetic retinopathy, Intraocular, Fenofibrate, TZDs, PPARs

## Abstract

**Background:**

Systemic therapeutics targeting the peroxisome proliferator-activated receptors have been found to be beneficial in the treatment of diabetic retinopathy. In this paper, we provide a rationale for the use of these therapeutics as intraocular agents. In addition, we introduce the peroxisome proliferator-activated receptors and describe their functions in response to the drugs.

**Discussion:**

Based on the evidence of large-scale clinical studies investigating the systemic administration of fenofibrate, this ligand for peroxisome proliferator-activated receptor-α is potentially a good candidate for intraocular delivery. Here, we describe the mechanisms by which it might be acting to improve diabetic retinopathy, its relative safety and we speculate on how it could be developed for intraocular delivery.

**Summary:**

In this paper, we provide a rationale for the further investigation of peroxisome proliferator-activated receptor-α agonists as intraocular agents for the treatment of diabetic retinopathy.

## Background

Diabetic retinopathy (DR) is a leading cause of blindness in adults, with some degree of DR occurring in nearly all type 1 diabetics and in the majority of type 2 diabetics [1]. Hitherto, it was thought that the only way to prevent the development and progression of DR was by tight control of blood sugar, plasma lipids and blood pressure [1]. However, it has since been shown that treatment of type 2 diabetics with lipid-lowering fenofibrates results in a significant reduction in the progression of DR which may be unrelated to any effect on plasma lipid levels [2,3]. A related class of compounds, the thiazolidinediones (TZDs), have been found to reduce progression to DR in at least one clinical trial [4]. Thus far, the beneficial effect of PPAR agonists on the retina has been observed following systemic delivery for the treatment of diabetic patients. It is our contention that the intraocular delivery of these agonists could specifically ameliorate DR.

**Figure 1:**
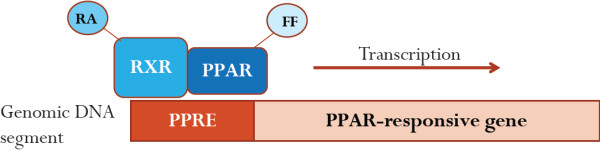
**Agonist-bound PPARs induce gene expression.** Activated PPARs must first associate with their co-receptor, retinoid X receptor (RXR), in order to modulate transcription of specific genes. RXR binds to its ligand, retinoic acid, and interacts with PPAR bound to an agonist (e.g. fibrates). Together, RXR and PPAR can then bind to a consensus sequence of nucleotides, known as the PPAR response element (PPRE). PPAR and RXR binding triggers expression of a responsive gene . Abbreviations: RA, retinoic acid, FF, fenofibrate.

Fibrates and TZDs are agonists for two isoforms of nuclear receptor superfamily members, the peroxisome proliferator-activated receptors (PPARs) α and γ, respectively. PPARs are localised to the nucleus, where they interact with other proteins involved in gene expression, including various co-activator and co-repressor proteins [5]. In order for PPARs to induce gene expression, they must also interact with their co-activator, the retinoic X receptor (RXR), and then bind to a PPAR response element (PPRE) in a given gene Figure 1[6-9]. PPARs modulate the expression of numerous genes, including those involved in lipid metabolism, adipogenesis, inflammatory signalling and oxidative stress [9-11], in a tissue-dependent manner [12]. Importantly, PPARs also regulate the expression of themselves via a positive feedback mechanism [13,14] or through a co-operative system with other transcription factors [15,16].

PPARs are generally considered to be inactive until bound by a ligand [17]. Both endogenous ligands and synthetic agonists have been described for PPARα and γ. Endogenous ligands include fatty acids and lipid metabolites, such as prostaglandins and leukotrienes [18-21]. Interestingly, prostaglandins and leukotrienes are also known to be mediators of inflammation and oxidative stress [22,23]. Synthetic agonists for the different PPAR isoforms are similar in terms of chemical structure and molecular mass; in fact, there are several known dual agonists that activate both PPARα and PPARγ [24,25]. The aforementioned synthetic PPAR agonists, fibrates and TZDs, are structurally very similar. However, research shows that fenofibrate specifically acts via PPARα [26], whilst TZDs are known to specifically activate PPARγ [27-30]. Further, PPARα and PPARγ are also known to have distinct physiological roles [9,31]. While PPARα increases the uptake and beta-oxidation of fatty acids, as well as reduces the synthesis and secretion of triglycerides, PPARγ specifically induces adipogenesis and stimulates triglyceride storage [12]. PPARγ is thought to improve insulin sensitivity via its stimulatory effects on GLUT4 and adiponectin [31]. Although both PPARα and PPARγ have been found to inhibit inflammation, they do so within different cell types via distinct targets [10,12]. Compared to PPARα, PPARγ has broader anti-inflammatory activity due to targeting numerous transcription factors within more cell types than PPARα [12]. In particular, PPARγ has been found to function as an inhibitor of monocyte/macrophage function by blocking pro-inflammatory signals [32-34]. To date, PPAR agonists have been clinically utilised for the treatment of diabetes and dyslipidaemia due to their beneficial effects on insulin sensitivity and lipid metabolism.

The PPARγ agonists, TZDs, are prescribed for lowering blood glucose levels [35] but it is uncertain whether they could also reduce DR progression since there are no randomised clinical trials. However, there has been one retrospective review of diabetic patients receiving rosiglitazone which revealed a reduction in development of proliferative DR [4], although this paper was criticised because of unmatched controls [36]. TZDs have also been trialled in animal models and were found to reduce choroidal neovascularisation [37]. Interestingly, the TZDs in this study were given by intraocular injection, suggesting that this delivery route of PPAR agonists might also be efficacious in humans [37]. Against the use of TZDs in the treatment of DR are the findings that systemic administration of troglitazone in humans was associated with increased vascular endothelial growth factor (VEGF) expression [38] and with an increased risk of diabetic macular oedema (DMO) [39]. Although the latter has been challenged by subsequent studies [40], questions remain about the overall safety and efficacy of TZDs [9,41,42]. In addition to TZDs, newer PPARγ modulators are being developed which could have enhanced safety profiles [9,43]. Further scientific and clinical studies are needed to clarify the role of PPARγ in DR and to determine whether treatment with TZDs or novel PPARγ agonists would be beneficial. Currently, no PPARγ agonists are known to reduce DR progression to the same extent observed with fibrates.

## Discussion

In the UK, under the NICE guidelines, fibrates are prescribed as a first-line therapy for diabetics with high serum triglycerides and are often given in combination with statins [35]. Recently, two large, randomised clinical trials showed an important secondary benefit of systemically-delivered fenofibrate on DR in type 2 diabetics. In the FIELD study, fenofibrate (200 mg/day) taken over five years reduced the need for laser photocoagulation to treat diabetic maculopathy by 36% and proliferative retinopathy by 32% [2]. Similar results were observed in the ACCORD Eye Study, wherein the use of fenofibrate along with simvastatin reduced progression of DR by 40% compared to simvastatin alone [3], with DR progression defined as a deterioration by three steps on the ETDRS severity scale. Importantly, in the FIELD study in particular, this benefit was independent of plasma lipid levels [2]. In addition, in both the FIELD and ACCORD studies, the benefit of fenofibrate was independent of glycaemic control. These findings could suggest that fenofibrate is having local effects within the eye not necessarily related to systemic metabolism.

Diabetic retinopathy is characterised by microangiopathy, which is thought to be caused by oxidative stress, advanced glycation end-products (AGEs), inflammatory mediators and endothelial cell death [44-46]. The beneficial effects of fenofibrate observed in the FIELD and ACCORD Eye studies could be due to reduced oxidative stress and inflammation, as well as effects on vascular function. Several studies have analysed the pharmacological mechanisms of fenofibrate individually. For example, fenofibrate has been shown to reduce circulating markers of oxidative stress in dyslipidaemic patients [47]. It has also been found to prevent inflammation by blocking AGE-induced NF-κB activation in animal models [48]. Fenofibrate has been found to ameliorate vascular function, improving blood flow in diabetics [49]. One recent study investigated the combined effects of fenofibrate on oxidative stress, inflammation and vascular tone in an animal model of diabetes [50]. This study found that fenofibrate improved vascular relaxation and increased expression of the antioxidant enzymes, superoxide dismutase and catalase [50]. Interestingly, they also observed a decrease in the level of a proinflammatory marker, myeloperoxidase (MPO) [50]. Importantly, a comprehensive screen of donated human retinal pigment epithelia (RPE) revealed that PPARα (the receptor for fenofibrate) was highly expressed while PPARγ was absent from the RPE [51]. Further, laboratory studies using human RPE cells under hyperglycaemic conditions found that fenofibrate reduced RPE monolayer permeability [52] via blocking activation of AMP-activated protein kinase (AMPK) [53,54] and the reduction in permeability was dose-dependent, indicating that intraocular delivery of fenofibrate could be highly efficacious. In summary, there is an expanding molecular basis for the positive effects of fenofibrate observed in the FIELD and ACCORD Eye studies.

Compared to other currently available PPAR agonists, fenofibrate appears to have a better safety profile. Fenofibrate has been prescribed for many years and is generally well-tolerated, with only 2% of patients discontinuing use due to side-effects [55]. There have been concerns that long-term use of fenofibrates might be associated with an increased risk of cardiovascular disease, particularly in those with renal impairment [56]. However, assessment of data from the FIELD study did not support this, providing further evidence for the safety profile of fenofibrate [56]. Given the findings of the large-scale FIELD and ACCORD Eye studies, fenofibrate has been found to reduce progression of DR and has not been associated with drug safety issues, making it a good candidate for potential intraocular delivery.

To our knowledge, the intraocular delivery of fenofibrate has not yet been examined. Therefore, animal and clinical trials are needed to determine if this delivery method would be suitable for the prevention and treatment of DR. The pharmacokinetics of these agonists in the eye is unknown. To date, fenofibrate has been formulated for oral delivery and is converted by esterases into the active compound, fenofibric acid [57]. Fenofibric acid has a half-life of 16 h and reaches a steady-state level in the circulation within five days of the commencement of treatment [55]. Given this need for esterase conversion, the delivery of a pre-activated form of fenofibrate into the eye could be more effective. One example of such a fenofibrate derivative is ABT-335, which is a choline salt of fenofibric acid and does not require esterase processing [55]. Such a readily bioactive drug could be better suited to intraocular delivery than the fenofibrate parent compound.

Should treatment with fenofibrate alone prove beneficial in the management of DR, combination therapies could then be examined. For example, it is now widespread practice to treat DMO with intraocular injections of anti-VEGF therapeutics [58-62]. While treatment with anti-VEGF is effective, it requires frequent injections [58,62-64]. This represents a burden on the patients and the healthcare delivery services. It is conceivable that intraocular anti-VEGF therapies could be combined with fenofibrate in the same injection, which might reduce the frequency of injections and therefore represent an improved treatment strategy. Whether the combination of fenofibrate and anti-VEGF agents will be viable for co-administration would need to be examined.

The goal of this paper is to provide a rationale for the intraocular injection of PPAR agonists, particularly fenofibrate or its derivatives, and to encourage further research in this area.

## Summary

· The ACCORD and FIELD Eye studies showed a significant beneficial effect on DR in diabetics treated with systemic fenofibrates.

· There is evidence from one study that intraocular delivery of TZDs is effective in treating CNV in an animal model, suggesting that PPAR agonists can be biologically active in the eye.

· Relative expression levels in the RPE suggest that PPARα agonists might be more beneficial than PPARγ agonists for the treatment of DR.

· There is emerging molecular evidence for the beneficial effects of PPARα agonists in the treatment of DR that goes beyond an improvement in plasma lipid levels.

· The commonplace treatment of DR with intraocular anti-VEGF agents could be used to facilitate PPARα agonist delivery. In other words, fibrates could be delivered concurrently with anti-VEGF using the same injection.

## Abbreviations

AGE: Advanced glycation end-products; CNV: Choroidal neovascularisation; DMO: Diabetic macular oedema; DR: Diabetic retinopathy; NF-κB: Nuclear factor-κB; PPAR: Peroxisome proliferator-activated receptor; PPRE: PPAR response element; RPE: Retinal pigment epithelium; RXR: Retinoic X receptor; TZDs: Thiazolidinediones; VEGF: Vascular endothelial growth factor.

## Competing interests

The authors declare that they have no competing interests.

## Authors’ contributions

The authors contributed equally to the analysis, design and writing of the article.

## Authors’ information

Dr. Maxwell Treacy is an engineer and trainee in ophthalmology. Dr. Tara Hurst is a biochemist, specialising in the innate immune response and viral modulation of cellular pathways.

## Pre-publication history

The pre-publication history for this paper can be accessed here:

http://www.biomedcentral.com/1471-2415/12/46/prepub
